# Does PBL deliver constructive collaboration for students in interprofessional tutorial groups?

**DOI:** 10.1186/s12909-019-1802-9

**Published:** 2019-09-18

**Authors:** Endang Lestari, Renée E. Stalmeijer, Doni Widyandana, Albert Scherpbier

**Affiliations:** 1 Medical Education Unit, Faculty of Medicine, Universitas Islam Sultan Agung, Semarang, Indonesia; 2 School of Health Professions Education, Faculty of Health, Medicine and Life Sciences, Maastricht University, Maastricht, The Netherlands; 3 Department of Medical Education, Faculty of Medicine, Gadjah Mada University, Yogyakarta, Indonesia

**Keywords:** Interprofessional problem-based learning, Maastricht peer-activity rating scale (MPARS)

## Abstract

**Background:**

Training health professional students in teamwork is recognized as an important step to create interprofessional collaboration in the clinical workplace. Interprofessional problem-based learning (PBL) is one learning approach that has been proposed to provide students with the opportunity to develop the necessary skills to work collaboratively with various health professionals. This study aimed to explore the extent to which students in interprofessional tutorial groups demonstrate constructive collaboration during group discussions.

**Methods:**

Students (*N* = 52) from the Medical, Midwifery and Nursing programmes took part in the study. Video-recordings were made of interprofessional PBL discussions (*N* = 40) in five groups, eight videos per group. Over a period of 4 weeks, participants discussed four scenarios concerned with the reproductive system. The resulting 67 h of video data were analysed qualitatively. To ensure inter-rater reliability, two tutors assessed the students’ constructive, collaborative activities using the Maastricht Peer-Activity Rating Scale (MPARS). Finally, to gain an understanding of students’ perceptions of their performance and participation in the interprofessional PBL tutorial, we organized three uni-professional focus groups (FGs) at the end of pilot project.

**Results:**

The translated MPARS was reliable (Kappa coefficient 0.01–0.20 and *p* < 0.05). Students were actively involved in the discussion and contributed to a better understanding regardless of their professional background. Group members from different professions complemented one another in solving learning issues. They were open, feeling free to question and argue from the viewpoint of their own profession, and also understood their strengths and limitations. The statistical test of the scores for constructive and collaborative activities indicated a significant difference between students and the various healthcare professionals, *p* = 0.000, with medical students scoring highest on both activities. Focus groups further clarified some of the observed dynamics.

**Conclusion:**

Implementing interprofessional PBL could motivate students to engage collaboratively in co-constructing knowledge to solve the patients’ problem. Medical students scored highest on constructive and collaborative activities.

## Introduction

Interprofessional collaboration in health care is considered to be a potential solution to reduce clinical error, improve patient safety and enhance the quality of patient care. Previous studies have shown that problems in collaboration and coordination between professionals can negatively affect patient outcomes, lower work satisfaction for professionals, and lead to wasted resources [1–7]. Previous studies report many factors that may prevent effective collaboration among professionals. These include professional factors, such as lack of knowledge about and trust in other professionals’ skills and expertise, and lack of understanding of the roles of other professionals [1, 8, 9] as well as external factors, such as professional culture, views, time constraints, problems contacting other professionals, and lack of reimbursement for collaborative work [9, 10].

Training health professional students to work together is recognized as an important step in creating interprofessional collaboration in the clinical workplace. Globally, the WHO supports health professions education to implement interprofessional education (IPE) [11]. IPE brings students from different health professions together to learn with, about and from each other, either in a classroom or a clinical setting [12–14]. IPE has been implemented in various educational formats, such as interprofessional ward-based training [15, 16], case-based discussion [17, 18], clinical simulation [19, 20], e-learning [21], and ambulatory primary care [22]. The key to effective learning in these interprofessional education programmes seems to be student interaction [23, 24]. Therefore, simply conducting shared lectures for students from different healthcare professions is unlikely to foster the attitudes and knowledge conducive to effective interprofessional teamwork [25]. Effective IPE should be interactive, collaborative, reflective, and experiential [26] and should strive to address the power relations and conflict inherent in health professional teamwork [27, 28].

Interprofessional problem-based learning (PBL) is one approach that has been proposed to provide students with the opportunities to develop the necessary skills to work collaboratively with different health professionals [29, 30] and effective learning approach for gaining in knowledge [31]. PBL is experiential, reflective, and intended to be interactive [32]. It provides opportunities to discuss, argue, present and hear one another’s viewpoints, thus contributing to the intellectual growth of students [33]. Interprofessional PBL could result in students developing the mutual professional respect and trust that is essential in interprofessional patient-centred practice. Essential for effective PBL is that students actively construct and reconstruct their knowledge in the group by summarizing, asking critical questions and correcting misconceptions [34–37] and that students actively collaborate in the process [38].

To date, research into interprofessional PBL has, among others, explored: student satisfaction while taking part in interprofessional PBL [39, 40], collaborative behavior within knowledge development [41], interprofessional attitudes pre and post interprofessional PBL [42], and students’ perceptions toward interprofessional PBL [31, 43]. However, whether the working ingredients of PBL, such as constructive and collaborative activities, still work when PBL is done within an interprofessional learning setting remains to be evaluated. The literature also reports that status factors and learners’ backgrounds affect interactions in small groups and thus the effectiveness of the group which, accordingly, affects productivity in constructing knowledge [44]. The question is whether PBL is an appropriate learning approach for interprofessional groups of students. Although interprofessional PBL was designed to foster collaborative, active learning skills in students, little is known about how it works in practice. This study aims to further clarify the inner workings of interprofessional PBL [45] and focus on examining constructive and collaborative activities among undergraduates taking part in an interprofessional PBL tutorial.

In order to achieve the aim of this study, we developed the following research questions:

1. To what extent do students in interprofessional PBL groups demonstrate constructive and collaborative activities in the tutorial group discussions?
2. To what extent do these activities differ between students from different professional groups?
3. How do the students reflect on their performance during interprofessional tutorial group discussions?

## Method

### Context

In Indonesia, all undergraduate health professions programmes have introduced interprofessional collaboration skills to their core curricula. However, very few Indonesian universities have actually incorporated an IPE programme that facilitates collaborative learning in interprofessional student teams into their curriculum. Universitas Islam Sultan Agung officially implemented IPE in 2017, and since 2012 has conducted several pilot projects on IPE, including interprofessional PBL tutorials and simulations for medical, nursing and midwifery programmes. The objectives of the IPE pilot project were to improve students’ ability to collaborate, share and communicate patient information with different professionals as a member of a health care team and to present an appropriate treatment and care plan to address the patient’s social, psychological and economic conditions.

Three healthcare education programmes are involved in the IPE pilot project, namely medical, nursing and midwifery programmes. These programmes differ in length and duration of their pre-clinical and clinical phases. While the medical and nursing programmes all have five-year curricula, the midwifery programme spans only 3 yrs. The clinical phases start after three and half pre-clinical years (medicine), 4 yrs (nursing), and two (Midwifery). Midwifery and nursing students have early clinical exposure in their pre-clinical phase in the form of 2 months of midwifery and nursing practice in a hospital or public health centre. Medical students do not have any practical experience in their pre-clinical years other than skills practice in the lab with simulated patients and manikins. Learning in all programmes is uni-professional, meaning that students rarely collaborate with students from other healthcare disciplines, not even during clinical rotations. As a pedagogical approach, PBL has been applied in the curriculum of each program. Therefore students experience learning collaboratively in the uni-professional setting. However, they never experience sharing knowledge and expertise with students from another professional background. For the present pilot study we invited students in their final pre-clinical year of medical, nursing, and midwifery to participate.

### Interprofessional problem-based learning tutorial

Uni-professional PBL tutorials have been applied in the programmes since 2005, so students do not need to learn how to conduct tutorial discussions. The PBL tutorial applied in the health-related programmes of Sultan Agung Islamic University employs seven jump steps [46, 47].

**Table:** 

PBL seven jump steps	
Step 1. Identify and clarify unfamiliar terms presented in the scenario; the scribe lists the terms that remain unexplained after the discussion Step 2. Define the problem or problems to be discussed Step 3. Use “brainstorming” to discuss the problem(s), suggesting possible explanations on the basis of prior knowledge; students draw on each other’s knowledge and identify areas of incomplete knowledge Step 4. Review steps 2 and 3 and arrange explanations into tentative solutions; the scribe organizes the explanations and restructures if necessary Step 5. Formulate learning objectives; group reaches consensus on the learning objectives Step 6. Private study Step 7. Students identify their learning resources and share the results of private study with group	

Tutorial session 1 (T1), which lasted 100 min, started by presenting the groups of students with the problems of clinical scenario. Through the group discussions and using prior knowledge of the content of the scenario, students identified learning issues (steps 1–5). After the discussion, students independently researched the learning issues outside the classroom (step 6). Students were given 3 days for self-directed learning. In this step, students have to study the learning issues related to both their areas of expertise and general medical science. For example, students had to study both the management and pathophysiology of pregnancy bleeding, including (other) risk factors of pregnancy. In tutorial session 2 (T2), which also lasted 100 min, the students regrouped to share the results of their self-directed learning (step 7).

Four scenarios (one per week) in the area of the reproductive system provided the topics of discussion. The background of the medical cases was interprofessional health care in a public health centre and the cases were problems that were commonly encountered in rural public health centres. The scenarios were treating: (1) tuberculosis (TB) during pregnancy, (2) vaginal bleeding during pregnancy in a public health setting, (3) hyperemesis gravidarum and (4) normal labour in a public health centre. For learning outcomes of the interprofessional PBL program, see Table 1.

**Table 1:** Learning outcomes

Week 1 Topic: Tuberculosis in pregnancy After attending the small group discussion tutorial, students were expected to be able to: • Explain the signs, symptoms and diagnosis of TB in pregnancyExplain the diagnostic procedure for TB in pregnancy • Explain the pharmacodynamics and pharmacokinetics of TB drugs and their side effects for pregnancy • Explain the role and responsibility of each profession of the health care team in handling a case of TB in pregnancy in the public health centre.	
Week 2 Topic: Vaginal bleeding After attending the small group discussion tutorial, students were expected to be able to: • Determine the scientific basis relevant to the pathophysiological understanding of the occurrence of vaginal bleeding in the third trimester of pregnancy • Describe the ethology and risk factors for vaginal bleeding in the third trimester of pregnancy • Describe the symptoms, signs, complications and abnormality of vaginal bleeding in the third trimester of pregnancy • Explain the differential diagnosis of vaginal bleeding in the third trimester of pregnancy • Explain the treatment administered to stop the patient bleeding in a public health centre and what should be done to refer the patient to hospital • Explain the role and responsibility of each profession of the health care team in handling vaginal bleeding in the third trimester of pregnancy case in the public health centre.	
Week 3 Topic: Hyperemesis gravidarum After attending the small group discussion tutorial, students were expected to be able to: • Explain the signs of emergency in pregnancy • Explain how to provide first aid in cases of severe dehydration / hypovolemic shock based on evidence-based medicine • Explain the management of hyperemesis gravidarum • Explain the role of each health profession in managing emergency cases in a public health centre.	
Week 4 Topic: Normal labour After attending the small group discussion tutorial, students were expected to be able to: • Explain the signs of labour • Explain the complications of labour • Explain the roles and responsibility of health care team members in handling third stage of labour in a public health centre setting • Explain the steps of collaboration among health care team members in handling normal labour in a public health centre setting • Explain the resuscitation procedure for new-borns.	

### Research design

We applied an explanatory, sequential mixed methods design to answer the research questions [48]. First we collected quantitative data on students’ constructive collaborative activities in interprofessional PBL tutorials by observing the video-recordings and filling out a previously inter-rater reliability-checked Maastricht Peer-Activity Rating Scale (MPARS). The results of the scale were then used as input for qualitative data collection, which consisted of uni-professional focus group discussions aimed to understand the underlying reasons for students’ perceptions of the interprofessional PBL tutorial. We also explored the students’ perception of their own performance of constructive and collaborative activities during the interprofessional PBL tutorial.

### Quantitative data collection and analysis: MPARS

All the tutorial processes were video-recorded. The recorders were set in the corner of the room to minimize any disruption to the participants’ behaviour. To analyse the students’ behaviour, we recorded 40 interprofessional PBL discussions (eight videos per group), resulting in approximately 67 h of video data.

To evaluate students’ constructive, collaborative and motivational activities, Kamp [49] developed the Maastricht Peer-Activity Rating Scale (MPARS). Containing 14 items, this scale is intended for assessing peer behaviour (constructive, collaborative and motivational activity) by students in uni-professional PBL tutorial discussions. In the present study, two tutors evaluated only the constructive and collaborative activities recorded on the videos of the interprofessional PBL tutorials. The constructive activities scale evaluates skills in co-constructing knowledge, such as summarizing, drawing distinctions between main and side issues, asking critical questions, correcting misconceptions, and contributing to a better understanding of knowledge. The collaborative activities scale evaluates collaborative performance during the discussion, such as a student’s influence on group members, their responsibility to the group, their willingness to share information, and their commitment to the group. The MPARS scale was translated by means of a double back translation procedure to assess the consistency between the original and translated versions. This means that an English-Indonesian translator translated the English version of the questionnaire into Bahasa Indonesian, after which another translator translated this version back into English. The instrument uses a five-point Likert scale ranging from (1) completely disagree; (2) disagree; (3) neutral; (4) agree; and (5) completely agree.

MPARS as a peer assessment tool has never been used before in Indonesia, or in any other Asian context. We felt that, as a measuring tool carried out by peers, MPARS; like other peer assessment tools might create too many feelings of discomfort in a cultural setting where saving face and speaking up are not self-evident [50, 51] Considering the characteristics of Asian students who might be biased in conducting peer assessment, in contrast to previous studies using MPARS, in this study the evaluation of students’ performance using MPARS was conducted by tutors rather than by students. Future research needs to explore how MPARS can be used as an effective peer-assessment tool in Asian settings. One of the researchers and a second ratter (junior tutor) assessed the constructive and collaborative activities recorded on videos of the interprofessional PBL tutorial to determine inter-rater reliability of the MPARS scale. Prior to the evaluation, the researchers and raters agreed on the evaluation items, so no differences in giving scores was expected. The evaluation results were collected and statistically tested with the Kappa test to determine the reliability of each item. The reliability and validity test was conducted employing SPSS (IBM SPSS Statistic).

Any differences in performing constructive and collaborative activities between students from each profession (medical, nursing and midwifery) were evaluated based on the average MPARS-item score, employing the Kruskal-Wallis test followed by the Mann-Whitney U statistical test.

### Qualitative data collection and analysis

#### Verbatim transcripts of the tutorial group meetings

To explore students’ actual engagement in interprofessional PBL tutorial groups, conversational data of the tutorial sessions were transcribed. The verbatim transcripts were made in Indonesian and coded for content, applying the coding scheme based on Kamp’s interaction analysis model [49].

The constructive and collaborative activity was evaluated from the discussion process. For the analysis we selected segments from the discussion of prior knowledge (step 3) in T1 and from sharing the results of self-directed learning (step 7) in T2, as in these steps the students discuss, share, argue, and present knowledge.

#### Focus groups

To gain a better understanding of students’ perceptions of their performance and participation in interprofessional PBL tutorials, we organized three uni-professional focus groups (FGs) at the end of pilot project. We deliberately chose not to mix students from different programmes to overcome potential barriers to communication and to encourage participation in the discussion. The focus group discussions were also video-recorded. A lecturer in community medicine (SY) and a medical educationist (DRA) who understood the concept and aims of the study facilitated the FGs with the aid of a discussion guide [52]. The purpose of this guide is to focus the discussion on the topic to be explored. The discussions were transcribed by an expert, and the verbatim transcript was coded for content without eroding the original content. Two experts in medical education EL and SY performed the thematic analysis. They independently evaluated the transcripts, first by open coding, and then developed and agreed on the coding categories, which they finally applied to the data. After this process, all members of the research team discussed the findings until they reached consensus. The data were analysed utilizing ATLAS.ti (version 7).

### Participants

Students in their final year medical, nursing and midwifery programmes voluntarily participated in mixed profession tutorial groups consisting of 8–10 students (Table 2).

**Table 2:** Group participants

Group	Profession of Tutor	Number of Medical students	Number of Nursing students	Number of Midwifery students	Total participants
Group 1	Nurse	3	4	3	10
Group 2	Nurse	4	4	3	11
Group 3	Doctor	3	5	3	11
Group 4	Doctor	3	5	2	10
Group 5	Midwife/ Doctor	3	4	3	10
Total		16	22	14	52

## Results

A total of 52 students from midwifery, nursing and medicine took part in the study (Table 3). Some students were absent for the discussions, particularly in the second, third and fourth weeks, due to other academic or non-academic commitments.

**Table 3:** Demographic characteristics of the participants

	Midwifery		Nursing		Medical	
	N	%	N	%	N	%
Gender						
Male	0	0	10	45.5	6	37.5
Female	14	100	12	54.5	10	62.5
Admission						
scholarship	1	7.1	0	0	1	6.3
regular test	13	92.9	22	100	15	93.7
Decision to study at the program						
own preference	14	100	18	81.8	14	87.5
encouraged by parents	0	0	4	18.2	2	12.5
Experience in collaborating with students from other departments						
Yes	10	71.4	12	54.5	12	75
No	4	28.6	10	45.5	4	25
	Mean	SD	Mean	SD	Mean	SD
Age	19.8	0.63	19.8	0.42	20.2	0.66
GPA (max score 4)	3.14	0.39	2.98	0.26	3.3	0.48

### MPARS inter-rater reliability and validity tests

The Kappa statistical test results indicated that all items had slight agreement with a Kappa coefficient of 0.01–0.20 and *p* < 0.05 (Table 4). The result of validity test indicated that all assessment items were valid to measure students’ co-construction and collaboration activities. The coefficient of corrected item-total correlation of all items were higher than 0.266 (correlation coefficient for 52 subjects).

**Table 4:** Inter-rater reliability of MPARS

		Reliability		Validity
No	Constructive activity	Kappa	P	corrected item- total correlation
1	Students were able to make adequate summaries	0.147	0.000	0.85
2	Students were able to make a distinction between the main and side issues in the subject matter	0.157	0.000	0.77
3	Students asked critical questions	0.078	0.020	0.77
4	Students corrected misconceptions about the subject matter	0.156	0.000	0.78
5	Students contributed to a better understanding of the subject	0.094	0.026	0.72
	Collaborative activity			
6	Students had a positive influence on the group	0.154	0.000	0.83
7	Students felt responsible for the group	0.108	0.000	0.75
8	Students promoted collaboration between group members	0.057	0.018	0.65
9	Students were willing to share their information	0.179	0.000	0.84
10	Students were committed to the group	0.046	0.047	0.71

### Constructive activities

Results indicated that medical students performed better on constructive activities than midwifery and nursing students. The result of the Kruskal-Wallis statistical test on all items of constructive activities indicated a significant difference in the constructive scores of students from different healthcare professions, *p* = 0.000 (Table 5).

**Table 5:** Constructive activities

Items	Midwifery		Nursing		Medical		p Kruskal- Wallis
	Mean	SD	Mean	SD	Mean	SD	
Constructive activity							
Students were able to make adequate summaries	2.48	0.48	2.59	0.55	3.22	0.39	0.000
Students were able to make a distinction between the main and side issues in the subject matter	2.94	0.53	2.89	0.50	3.22	0.31	0.000
Students asked critical questions	2.66	0.63	2.59	0.43	3.05	0.40	0.000
Students corrected misconceptions about the subject matter	2.62	0.54	2.59	0.43	3.05	0.40	0.000
Students contributed to a better understanding of the subject	2.69	0.53	2.89	0.57	3.43	0.39	0.000

Mann-Whitney testing between each of the two groups indicated that for all scale items, the mean score of midwifery and nursing students was not significantly different (*p* > 0.05). Meanwhile, in all assessed items, there was a significant difference in the scores of medical students with that of midwifery students and nursing students (*p* < 0.05).

Based on the analysis of the videos and transcripts of the tutorial group meetings, students were actively involved in the discussion and contributed to a better understanding regardless of their professional background. However, depending on the topic, we saw differences in the extent to which different groups of students engaged. Medical students contributed the most in the discussion of physiology, pathophysiology and clinical reasoning to decide on a diagnosis or a differential diagnosis but less in management. Midwifery students also contributed to elaborating knowledge of physiology, pathophysiology, specifically pregnancy, and they were best in explaining the management and treatment for normal pregnancy, but they participated less in patient management other than normal pregnancy. Meanwhile, nursing students were very active in elaborating information when the topic concerned practical management and treatment of the patient, but were less active in the discussion of physiology, pathophysiology and clinical reasoning to decide a diagnose or a differential diagnose. Group members who did not answer questions or explain knowledge became active listeners. This could be observed from the fact that they paid close attention to the other group members’ conversation, asked clarifying and probing questions, added further information, rephrased or summarized to check their understanding, and waited until a group member had done speaking before responding.

Quotes from discussions:

*“In the case of inducing labour when the fetal heart rate is abnormal, we usually administer oxygen by mask and lay the mother on her side.” (Midwifery student 3)*

*“Why she should be treated with oxygen and laid on her side?” (Nursing student 5)*

*“When she’s lying on her side, I think it’s easier for the nutrients to enter the fetus.” (Midwifery student 3)*

*“It just has to do with technique.”(Midwifery student 1)*

*“I learned there are a few possible labour positions. One of them is lying on her side. The mother lies on her left or right side with one leg raised, and the other leg straight… The benefit of this position is that it reduces pain in the waist, helps lower high blood pressure, and accelerates the labour process. This position makes the blood delivery from mother to fetus run well through the placenta and then labour is more comfortable.”(Medical student 4)*

### Collaborative activities

The result of the Kruskal-Wallis statistical test for all items on collaborative activities indicated a significant difference between the MPAR scores of students from different healthcare professions, *p* = 0.000. The Mann-Whitney test between each group pointed out that for all scale items, the mean scores of midwifery and nursing students were not significantly different (*p* > 0.05). Meanwhile, in all assessed items, there was a significant difference in the mean score of medical students with that of midwifery students and nursing students (*p* < 0.05) (Table 6).

Students encouraged and facilitated one another when they discussed the learning issues. Group members from different professions complemented others in answering learning issues. They were open to each other, feeling free to ask and argue their professional viewpoints, and also understood their limitations and strengths. In addition, the role of group leader switched from profession to profession. The leader stimulated shared responsibility for the learning in the tutorial groups and helped the discussion run smoothly.

**Table 6:** Collaborative activities

Items	Midwifery		Nursing		Medical		p Kruskal- Wallis
	Mean	SD	Mean	SD	Mean	SD	
Collaborative activity							
Students had a positive influence on the group	3.12	0.39	3.22	0.5	3.54	0.34	0.000
Students felt responsible for the group	3.35	0.53	3.47	0.48	3.66	0.25	0.002
Students promoted collaboration with group members	3.37	0.29	3.42	0.40	3.64	0.31	0.000
Students were willing to share their information	3.11	0.55	3.11	0.52	3.68	0.28	0.000
Students were committed to the group	3.37	0.49	3.24	0.38	3.64	0.24	0.000

Collaboration was also apparent when students in one group of various professionals helped one another find answers and solve problems instigated by the tutors’ critical questions.

*“… It’s important to know that pregnant women get the same TB treatment as other TB patients. Pregnant women can take rifampicin, isoniazid, ethambutol, and pyrazinamide all safely. There are indeed side effects of the drugs, both mild and severe….” (Medical student 1)*

*“Pregnant and non-pregnant women get the same treatment?” (Tutor)*

*“… Except for streptomycin … Pregnant women should not be given streptomycin because of its ototoxicity. It causes calcium levels to drop in the blood and extreme loss of body water so it’s harmful to the fetus.”(Medical student 1)*

*“That’s for category 1 and 2 so this combination is for two-month treatment. After that the patient should undergo another sputum smear.” (Nursing student 4)*

The above example of collaboration indicates that conflict on conceptual knowledge can be resolved collaboratively among professions.

### Qualitative findings

To address the research question ‘how do the students reflect on their performance during interprofessional tutorial group discussions?’ and to allow for a better understanding of the interprofessional PBL process, we organized uni-professional focus groups. Five main themes were identified, specifically: 1) Students learned from each other professions’ knowledge, 2) asking critical questions is not always self-evident 3) correcting misunderstandings without causing offence 4) Factor affecting students participation, 5) persisting professional barriers.

1. **Students learned from each other professions’ knowledge.**

During the focus group discussions, students said that they benefitted from the differences in the knowledge of each professional group, and that they were able to both provide and gain knowledge. Furthermore, it helped them understand the limitations of their own profession.

“*It’s good to meet students from different programmes. I learned a lot from other professions, like I learned the steps to handle emergency patients from the nursing students.” (Medical student 3)*

*“I learned how to apply clinical reasoning to diagnose patients from medical students” (Nursing student 2)*

Interestingly, the students discussed not only medically related topics, such as physiology, pathophysiology, diagnosis, and management but also the roles and responsibilities of each profession related to the cases. This enabled the students to learn about the boundaries between roles and also the limitations of their own role.

*“In IPE we learned what role each profession must play in collaborative healthcare. It’s important so that responsibility can be shared and the patient can be treated quickly and correctly” (Nursing student 4)*

2. **Asking critical questions is not always self-evident**

Some students asked critical questions, usually to broaden understanding or deepen the topic. Nevertheless, the posing of critical questions was strongly influenced by the role of the tutor who usually asked critical questions to challenge students and stimulate deep learning. However, in groups with a very dominant tutor, this person mostly posed the critical questions which consequently reduced the students’ role in such constructive learning activities as searching for links between topics and understanding mechanisms/theories by themselves. As a result, students tended to rely on the tutor’s questions to develop the concept.

***“Can a dead baby possibly be delivered spontaneously?” (Tutor)***

*“Do you mean the mother does not know that the baby has passed away?” (Midwifery student 5)*

***“Yes. Dead for months, for example. Can it still be delivered spontaneously?” (Tutor)***

*“I think the baby can be delivered spontaneously after it dies, but not [when it is dead] for as long as months, like you said.” (Medical Student 2)*

***“Have you ever heard of abortion?” (Tutor)***

*“Yes” (All students)*

*“W* ***hat are the complications of abortion?” (Tutor)***

Some students explained that asking questions was not nice for classmates, as the classmates then had to give further explanation and elaborate on the concept that they were trying to explain to the class. Asking questions should be avoided to maintain a conducive and comfortable discussion situation.

*“We loved adding information rather than asking for further explanation. We do that in uni-professional tutorial as well. It’s common for us students… asking questions means challenging our mates to explain. It’s putting a burden on them.” (Medical student 6)*

*“…we understand that asking ‘further questions or for clarification’ will broaden our knowledge and understanding, but as my friend said, it burdens our mates and gives them problems. That’s why we try to avoid it. We let our tutor ask the critical questions and bring those questions to the table so that all students are responsible for answering collaboratively.” (Medical student 2)*

Students pointed out that asking critical questions was also regarded as creating conflict, which would arise when students held different points of view. In that situation, it was apparent that students would come to a quick consensus and agree to avoid the inconvenient situation caused by differences.

*“Critical questions will only produce new problem to discuss, and will sometimes create conflict. We don’t like conflict. We love doing smooth discussion. That [avoiding conflict] makes us feel comfortable in the small group discussion.” (Midwifery student 1)*

*“Difference of opinion happens sometimes, but we don’t want to make it worse. For me, I’d rather accept another professional’s opinion, understand their point of view and try to compromise on the difference.” (Medical student 5)*

Moreover, the analysis of students’ activity during interprofessional tutorials indicated that the group leaders, students who could be from any profession, generally drew the conclusions of the discussion. Some groups drew no conclusions and the chair simply asked the group members whether all had understood and agreed with the discussion content. When all participants agreed with the explanation, the discussion continued on to the next topic. When we explored this phenomenon later on, the focus group students explained that it was common practice: if all the explanations were clear and there were no differences in opinion, then they would immediately agree and just go on to the next question.

*“When there are no conflicting views and all the explanation are clear and we agree with them, then don’t think we need to sum up.” (Nursing student 2)*

3. **Correcting misunderstandings without causing offence**

Correcting misunderstandings of the concept also occurred during interprofessional discussion. The interesting thing was that students tended to correct misunderstandings in students from other professions indirectly, in a polite manner, for example by quoting information from a learning resource they had read, rather than expressing direct disapproval [criticism].

*“OK, let’s expand the topic... If the fetus died in the womb, what should the health care team do?” (Tutor)*

*“C-section?” (Nursing student 3)*

*“Induction?” (Midwifery student 1)*

*“Do you mean per vagina?” (Tutor)*

*“If the fetus dies in the womb, it will come out by itself as the fetus will be considered a foreign body by the pregnant body.” (Midwifery student 2)*

*“I read in Achdiat 2004 that there are several ways to manage fetal death in the womb. Dilation or curettage can be administered for pregnancy less than 12 weeks gestation. For pregnancy over 12 weeks…” (Medical student 4)*

The focus group students explained that correcting misconceptions by providing information from learning resources was done to avoid causing offence to another group member.

*“It also happens with correcting mistakes. We correct misunderstandings in other profession students politely, by providing another perspective from medical resources. So we don’t say directly that the other person’s opinion is wrong. We try to be as polite as possible so that others won’t be offended. I don’t want to let other students in the other professions think that we, the medical students, are more powerful than them.” (Medical student 3)*

4. **Factors affecting students’ participation**

- The role of tutor

Some students in the focus group clarified the strong role of the tutor in constructive learning activities and mention that tutors were too active. However, some students said that they appreciated the tutor taking an active role.

“*Our tutor is so active. She asks a lot. But I think it’s good because it can expand the topic of discussion.” (Midwifery student 3)*

b. Social status

Another factor hampering constructive learning was the difference in social status of the health profession groups. According to the students, ‘inequality’ made them reluctant to criticize opinions and pose critical questions to other students.

*“Sometimes we feel too uncomfortable to ask [questions]. Embarrassed, I feel like I lack knowledge, especially [compared] to medical students.” (Midwifery student 5)*

5. **Persisting professional barriers**

We observed an interesting phenomenon with regard to students’ collaborative behaviour. Despite collaborating solidly in their interactions for several weeks, we still found professional barriers up until the last week of meetings. For example, students still clustered physically in accordance with their profession; especially midwifery students. When this was explored during the focus group, students said that the problem was closely related to confidence. Students felt secure when sitting beside a friend from the same profession so that they could discuss the answer to a problem based on their shared background of professional knowledge. Some students felt that the interprofessional class was quite stressful, because they had to maintain professional pride.

Bellow are some quotes from the focus group discussion that indicating insecurity during interprofessional PBL.

*“Yes… we always sit beside each other. We feel confident, and feel that we can support each other if we sit side by side. So, if we have problem, we can negotiate with the others according to our scientific background.” (Midwifery student 2)*

*“Sitting next to a student from the same background made us feel safe. The discussion was so tough for us, it forced us to struggle to do our best because we had to uphold the pride of our profession.” (Nursing student 4)*

## Discussion

Using a mixed methods design we set out to study how students engage in constructive collaboration in interprofessional PBL tutorial meetings, how the performance of each professional group differed and how students motivated their performance during the tutorials.

Based on the observations of the tutorials and using the MPARS, two researchers rated the students to distinguish those who very actively contributed to construct knowledge regardless of their professional background. These students collaborated on developing knowledge and complemented one another in answering the learning issues. They shared knowledge and learned about one another’s professions, including the role boundaries and limitations. These findings suggest that the PBL setting meets the aims of IPE – to experience the perspectives held by others, to listen to the way they talk about their tasks and competencies and to construct knowledge in collaboration with one another [43, 53, 54]. Students were observed to correct each other’s misconceptions interprofessionally. Very encouraging was the fact that corrections were voiced politely in non-confrontational language, indicating respect for the fellow student and potentially the other’s profession. These findings resonate with previously reported studies which describe that interprofessional PBL could inculcate respect for other professions and appreciation of the roles and knowledge of others [32, 55].

Our findings provide examples of collaborative interprofessional practice when it comes to solving the patients’ problem. Interestingly, conflict on conceptual knowledge can be elaborated collaboratively among the professions. Others have demonstrated how collaboration in PBL might have favourable outcomes for IPE because it helps in creating a more positive attitude towards other professional groups and improving interprofessional relations [32, 43, 53, 56, 57].

In addition to these promising findings, our results show that students often try to avoid conflicts in the discussion or, when conflicts arise, they accept sketchy arguments and conclude the discussion quickly. This could be problematic, as learning to cope with uncertainty is an essential goal in PBL and the ability to avoid hasty conclusions in uncertain situations is vital for future clinical practice [58]. However, these phenomena are also observed in uni-professional PBL [59]. Our findings imply that students’ discussion skills need to be enhanced, such as the skills required to bring out differences in each other’s conceptual thinking, to develop deep argumentation and to produce questions that elicit elaboration. The tutors’ ability to facilitate collaborative resolution of conflicts in knowledge in the interprofessional tutorial should be improved.

It was also found that midwifery and nursing students scored between poor and average on their constructive activities lower than medical students’ scores, pointing to unequal participation in the PBL sessions. Medical students also scored higher on their collaborative activities. The focus group findings shed light on factors hindering equal participation, such as cultural aspects, the students’ perception of hierarchy in the field of health services, and lack of self-confidence.

The role of cultural practices in relation to the success of PBL has been previously described [29, 60]. Active learning techniques, such as the PBL tutorial, have gained popularity at medical schools in western countries. However, there are problems to be faced in executing the method, particularly among Asian students who are used to gaining knowledge passively through didactic lectures, being spoon-fed and memorizing knowledge without criticizing itThe successful application of the PBL methods in Asian schools is impeded by different cultural practices, such as the students’ lack of confidence in sharing their opinions, reluctance to criticize and share a different point of view and their preference for classic, didactic lectures and memorizing facts rather than extracting problems from the cases by themselves [61, 62]. As a result, the benefits of PBL designed to train students to argue, criticize and co-construct knowledge are less than optimally achievable ([63, 64]. Our findings indicate that critical questions were seldom asked during the discussions. This could be caused by a combination of cultural and interprofessional factors. The resulting dominance of the tutor in these cases has also been previously reported [60, 65]. Facilitating IPE is complex and demanding, which makes the faculty development of tutors in an IPE setting critical [66–68].

Lessons to be learned from this research are that students from various professions can benefit from PBL interprofessional activities, such as being able to collaborate in constructing knowledge and practicing communicating with other professions. Also, IPE PBL could teach the student the importance of respecting and fostering respect for the roles of other professions, taking advantage of working in a team to tackle complex, difficult problems and discussing a patient-centred approach to care. This finding was in accordance with previous research which explored students’ perception regarding interprofessional education and reported that students were favourable to IPE [69]. However, in this study, interprofessional PBL did not succeed in creating more equality in the process as medical students were better at constructive and collaborative activities. This seems to be a result of the interplay between various complex factors, such as the influence of the Asian cultures that tend to be hierarchical and place doctors in higher positions in society, and problems with self-confidence and the students’ learning preferences. Considering these findings, it is suggested that PBL should not be the only learning approach applied to IPE. It can be as useful starting point for students from different professions in the pre-clinical year phase to interact in IPE, but then it should be followed by simulation and work-based learning approaches. The recent study by Paradis & Whitehead also suggests that interprofessional training only makes sense when applying practices in the workplace [28].

This study contributes to literature as it provides pedagogical implication through examining students’ actual performance and reflection on their participation in interprofessional PBL. The limitation of this study was that it was a small pilot project with a relatively small group of participants from three programs only. They might not represent the performance and perceptions of all Indonesian healthcare professional students. Moreover, students participating were volunteers so they may have had a stronger interest in experiencing IPE. Future research should include explorational and observational designs to study students’ performances within interprofessional PBL among large numbers of students.

## Conclusion

Implementing interprofessional PBL could motivate students to engage in the co-construction of knowledge and other collaborative activities to solve patients’ problems. However, because PBL is influenced by national and professional cultures, implementing PBL alone is probably not enough to achieve all the IPE goals. In the Asian context, we suggest that PBL should be followed by other learning approaches in the continuum of study in the professional health care curriculum. There was evidence from this study that MPARS was valid and reliable instrument to evaluate students’ constructive and collaborative activities during interprofessional PBL. Further research could implement the MPARS as a peer-assessment tool and help improve the tutorial group process.

## Data Availability

Materials and supporting data are available for download on the website: https://drive.google.com/drive/folders/1iTTTzCAJnXDKdrKHVWv7ZA2pOKtryRAb All files may be used for research and education without further consent.

## Abbreviations

FG: Focus group
FGD: Focus group discussion
GPA: Grade point average
IPE: Interprofessional education
KMO: Kaiser-Meyer-Olkin
MPARS: Maastricht Peer-Activity Rating Scale
